# Clinical thought-based software for diagnosing developmental dysplasia of the hip on pediatric pelvic radiographs

**DOI:** 10.3389/fped.2023.1080194

**Published:** 2023-03-30

**Authors:** Jia Sha, Luyu Huang, Yaopeng Chen, Zongzhi Fan, Jincong Lin, Qinghai Yang, Yi Li, Yabo Yan

**Affiliations:** ^1^ Department of Orthopedics, Xijing Hospital, Air Force Military Medical University, Xi’an, China; ^2^ School of Telecommunications Engineering, Xidian University, Xi’an, China; ^3^ Guangzhou Institute, Xidian University, Xi’an, China

**Keywords:** developmental dysplasia of the hip, children, pelvic radiograph, diagnosis, software

## Abstract

**Background:**

The common methods of radiographic diagnosis of developmental dysplasia of the hip (DDH) include measuring hip parameters and quantifying the degree of hip dislocation. However, clinical thought-based analysis of hip parameters may be a more effective way to achieve expert-like diagnoses of DDH. This study aims to develop a diagnostic strategy-based software for pediatric DDH and validate its clinical feasibility.

**Methods:**

In total, 543 anteroposterior pelvic radiographs were retrospectively collected from January 2017 to December 2021. Two independent clinicians measured four diagnostic indices to compare the diagnoses made by the software and conventional manual method. The diagnostic accuracy was evaluated using the receiver operator characteristic (ROC) curves and confusion matrix, and the consistency of parametric measurements was assessed using Bland-Altman plots.

**Results:**

In 543 cases (1,086 hips), the area under the curve, accuracy, sensitivity, and specificity of the software for diagnosing DDH were 0.988–0.994, 99.08%–99.72%, 98.07%–100.00%, and 99.59%, respectively. Compared with the expert panel, the Bland-Altman 95% limits of agreement for the acetabular index, as determined by the software, were −2.09°–2.91° (junior orthopedist) and −1.98°–2.72° (intermediate orthopedist). As for the lateral center-edge angle, the 95% limits were −3.68°–5.28° (junior orthopedist) and −2.94°–4.59° (intermediate orthopedist).

**Conclusions:**

The software can provide expert-like analysis of pelvic radiographs and obtain the radiographic diagnosis of pediatric DDH with great consistency and efficiency. Its initial success lays the groundwork for developing a full-intelligent comprehensive diagnostic system of DDH.

## 1. Introduction

Developmental dysplasia of the hip (DDH) is a common skeletal deformity in children and the prominent cause of hip osteoarthritis and lower limb disability (1, 2). Early diagnosis and timely treatment of DDH are beneficial to promote normal hip development (3–5). Anteroposterior pelvic radiography is the first-line screening examination for evaluating DDH in children over 4–6 months (4). Dynamic splinting, represented by the Pavlik harness, is the preferred therapeutic option for children under six months with reducible DDH; static bracing (e.g., the rhino brace) is for children over 6–9 months with DDH needing continued abduction positioning (6).

Improper interpretation of pelvic radiographs may misdiagnose pediatric DDH, impair the hip's development, and cause long-term joint dysfunction (7, 8). As machine learning developed, several scholars began trying to apply artificial intelligence algorithms to assist in diagnosing DDH (9–13). This technique is practical but still in its infancy (3). Its limitations include inaccuracy in locating key landmarks, absence of diagnostic criteria, and failure to achieve an integrated diagnosis by considering the correlation between parameter values and their clinical significance (9, 14–16).

However, clinical experts' diagnosis of DDH requires the patient-centered analysis and judgment of hip parameters by combining medical knowledge and personal information (i.e., clinical thought) (17–19). Additionally, the immaturity of pediatric hips leads to significant variation in the morphology of anatomical landmarks among children of different ages, especially in DDH (8, 20). Therefore, it is challenging to rely solely on machine learning to locate anatomical landmarks in children. The clinical diagnosis of DDH still relies primarily on manual labeling, measurement, and analysis methods.

This study developed and validated a diagnostic strategy-based software for DDH against the above issues, prioritizing accuracy while pursuing efficiency. To our knowledge, the proposed software was the first attempt to integrate the diagnostic strategy of pediatric DDH into the evaluation of anteroposterior pelvic radiographs. Firstly, the end-user manually annotated anatomical key points on pelvic radiographs. Then some points deviating from the bony rim were rectified based on the minimum Euclidean distance. Next, the key points' coordinate information was used to obtain hip parameters. Finally, an integrated diagnosis of the pelvic radiograph was made *via* the built-in diagnostic strategy for DDH. The hypothesis is that the software can achieve an expert-like diagnosis of pediatric DDH quickly and accurately.

## 2. Methods

### 2.1. Patients

The retrospective studies involving human participants were reviewed and approved by the Medical Ethics Committee of the First Affiliated Hospital of the Airforce Medical University. The requirement of informed consent was waived due to the anonymous use of imaging data. The original 1,606 anonymized anteroposterior pelvic radiographs from the Radiology department of Xijing hospital between January 2017 and December 2021 were included in the study with the following inclusion criteria: (1) age of 0.4–8.0 years; (2) available pelvic radiographs covering the area from the level of the iliac crest to the mid-superior femur; (3) children with chief complaints of hip pain or suspected DDH; (4) initial visit.

During pelvic radiography, the children were instructed in a standard anteroposterior supine position with both lower extremities internally rotated by 15° (21). The exclusion criteria for ineligible cases were radiographs with (1) extreme tilt or rotation according to Tönnis criteria (the rotation quotient or symphyseal-ischial angle exceeded the reference range) (22), (2) previous history of hip surgery, (3) traumatic or infectious hip deformities, and (4) other congenital hip deformities such as Legg-Calvé-Perthes disease. There were 543 radiographs finally incorporated into the study (Table 1).

**Table 1:** Patient demographics and radiographic diagnosis.

Parameter	Value
Cases (*n*)	543
Gender, F: M (*n*)	408:135
Mean age (years)	2.0 (95%CI 0.6–4.3)
**Age, *n***	
0–2 years	330
≥2 years	213
**Diagnosis (hips)**	
**Non-DDH**	723
Normal	715
Other	8
**DDH (IHDI classification)**	363
Grade 1	128
Grade 2	62
Grade 3	63
Grade 4	110

### 2.2. Built-in diagnostic strategy for DDH

**Figure 1:**
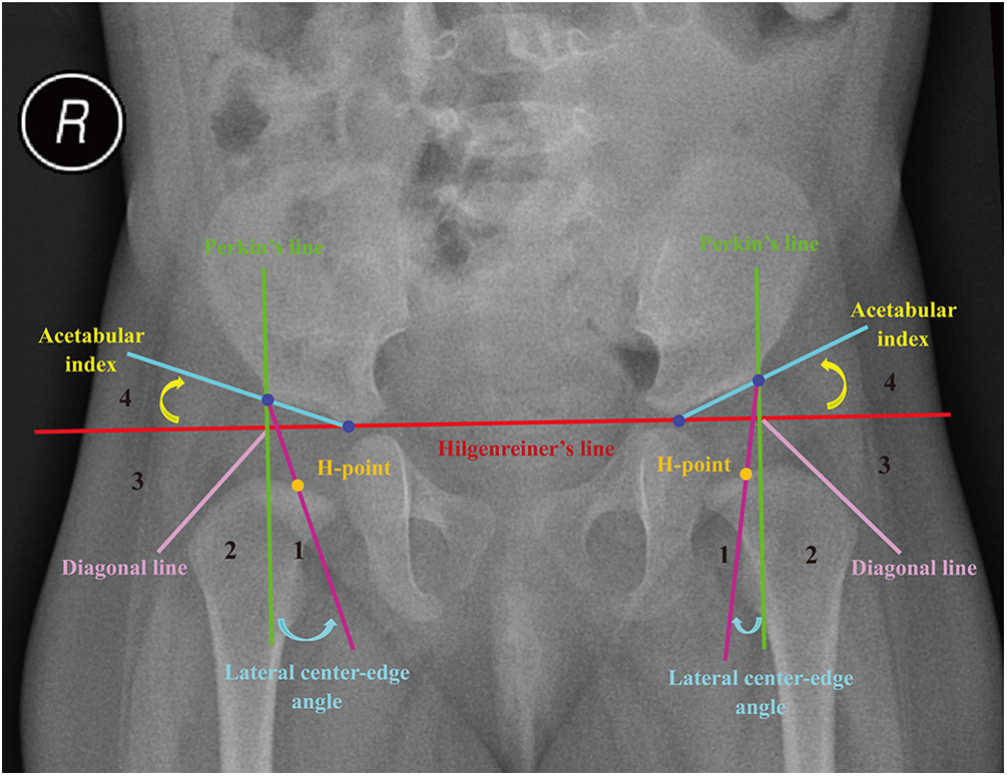
Diagnostic references of developmental dysplasia of the hip (relies on the midpoint of the proximal femoral metaphysis, H-point). Hilgenreiner’s line is a basal line connecting the top of bilateral triradiate cartilages. Perkin’s line is perpendicular to Hilgenreiner's line, passing through the lateral acetabular edge. The diagonal line is drawn 45 degrees from the junction of Perkin's line and Hilgenreiner's line. The acetabular index is the angle between Hilgenreiner's line and the line from the top of the triradiate cartilage to the lateral acetabular edge. Lateral center-edge angle is the angle between a vertical line through the center of the femoral head and a line connecting that center and the lateral acetabular edge.

In this study, the acetabular index of Hilgenreiner (AI) was used to assess acetabular development in children (23). Its normal range was influenced by the child's nationality, gender, age, and bilateral differences (17, 18). Meanwhile, Wiberg's lateral center-edge angle (LCEA) was chosen to assess the acetabular coverage (24). Pediatric DDH was diagnosed when AI exceeded double standard deviations from the mean $\left(\bar{x}+2s\right)$. Different criterias depending on the nationality (Zhao's and Tönnis' criteria for Chinese and non-Chinese, respectively) were adopted (17, 18). Furthermore, the International Hip Dysplasia Institute (IHDI) radiographic classification was used to quantify the severity of DDH (25–27). The relative position of the midpoint of the proximal femoral metaphysis (H-point) further classified pediatric DDH into IHDI grade 1 to 4 hips. Non-DDH were then divided into normal and other dislocated hips (e.g., neurogenic dislocation of the hip) according to the presence or absence of hip dislocation. The detailed diagnostic strategy for pediatric DDH is shown in Figure 1 and Table 2.

**Table 2:** The radiographic diagnosis for pediatric developmental dysplasia of the hip.

Diagnosis	Description
**DDH**	Abnormal acetabular index
IHDI 1	The midpoint of the proximal femoral metaphysis (H-point) is at or medial to Perkin's line.
IHDI 2	H-point is lateral to Perkin's line and at or medial to the diagonal line.
IHDI 3	H-point is lateral to the diagonal line and at or inferior to Hilgenreiner's line.
IHDI 4	H-point is superior to Hilgenreiner's line.
**Non-DDH**	Normal acetabular index
Normal	Non-dislocated hip
Other	Other hip dislocation (e.g., neurogenic dislocation of the hip)

The diagonal line is drawn 45 degrees from the junction of Perkin's line and Hilgenreiner's line.

### 2.3. Application implementation

**Figure 2:**
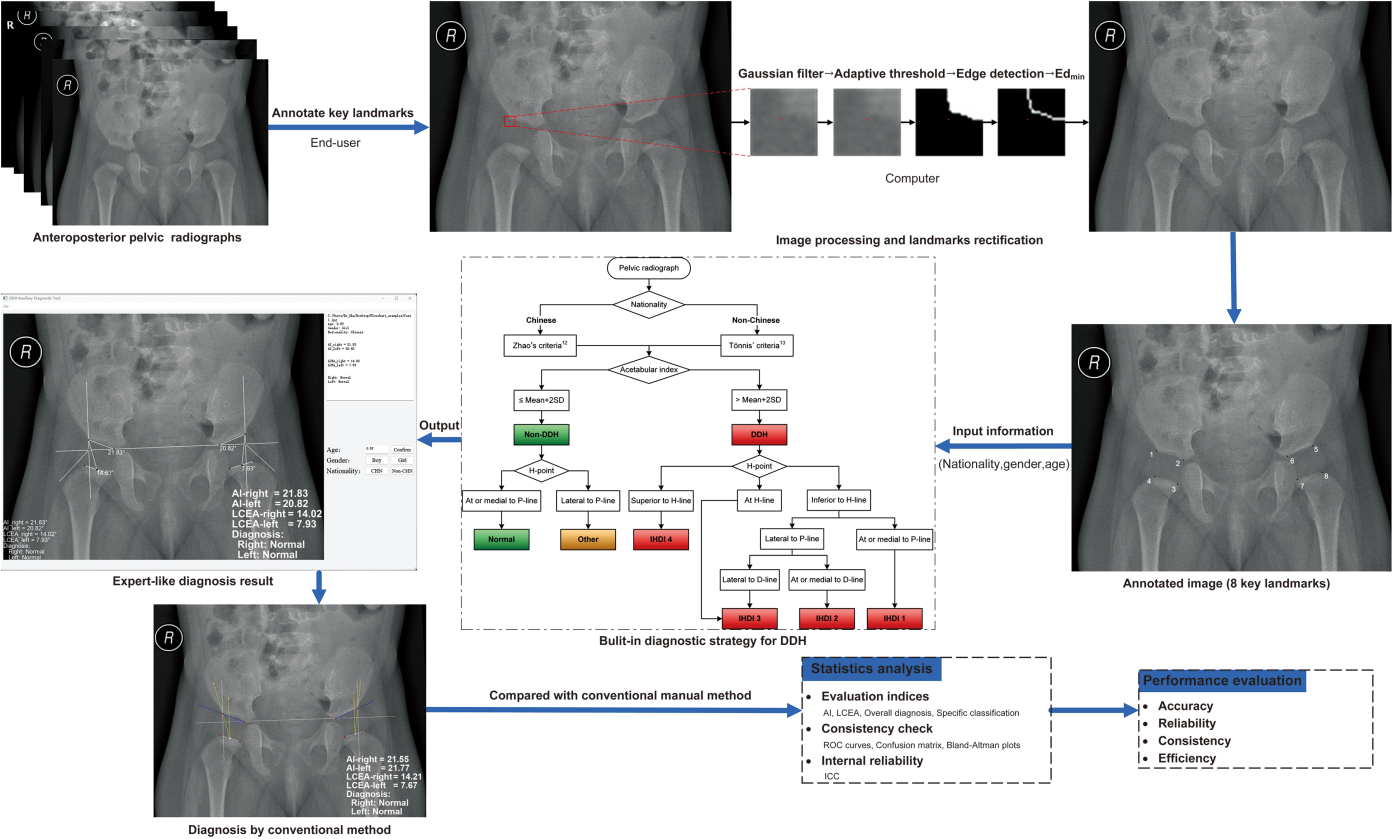
Detailed workflow of the proposed software for developmental dysplasia of the hip.

The proposed computer-aided software used a three-step approach to diagnose pediatric DDH from pelvic radiographs (Figure 2). For the first step, the end-user manually annotated anatomical key points on pelvic radiographs to obtain their initial coordinates (x, y). The key points included the lateral acetabular edge, the top of the triradiate cartilage, and the inner and outer points of the proximal femoral metaphysis. For some points deviating from the bony rim, the computer intercepted an area of 30 × 30 pixels around them and turned the area into a grayscale image. After the processes of Gaussian filtering, adaptive threshold, and edge detection, the bony rim was identified. Then the Euclidean distance (Equation 1) from the initial annotated point (x, y) to the rim was calculated. After that, the pixel point on the rim (a, b) that minimizes the Ed was selected as the rectified annotated point.

(1) $$\mathrm{Ed}=\sqrt{{\left(x-a\right)}^{2}+{\left(y-b\right)}^{2}}$$

In the second step, diagnostic references (e.g., the H-point, Hilgenreiner's line, and Perkin's line) were drawn based on the annotated points' coordinates. Then the acetabulum-head relationship and hip parameters were measured automatically. The third step was to input a child's general information (i.e., nationality, gender, and age) by the end-user to select the appropriate diagnostic criteria for DDH. Subsequently, an expert-like radiographic diagnosis on the pelvic radiograph was performed using the built-in diagnostic strategy of pediatric DDH.

### 2.4. Performance testing

The AI, LCEA, overall diagnosis (non-DDH and DDH), and specific classification (normal, other dislocated, and IHDI grade 1–4 hips) were calculated to evaluate the diagnostic performance between the software and conventional manual method. Two senior pediatric orthopedists and two senior pediatric radiologists were invited to form an expert panel. Using their undisputed diagnostic results as the standard, the other two independent orthopedists measured these indices to compare the performance difference between the two methods. The two orthopedists include an intermediate orthopedist (attending surgeon) and a junior orthopedist (resident surgeon). To evaluate intra-reliability, they measured each diagnostic index twice at 4-week intervals. Besides, the difference in time consumption between the two methods was compared.

The image analysis software Digimizer 5.7.2 (MedCalc Software, Ostend, Belgium) was used to simulate the conventional manual assessment of pelvic radiographs. The main steps were as follows: (1) marking the top of bilateral triradiate cartilage, the lateral acetabular edge, and H-point, (2) drawing Hilgenreiner's line, Perkin's line, and the diagonal line, (3) calculating the AI, LCEA, and IHDI classifications, (4) making final judgments.

### 2.5. Statistical analysis

The receiver operating characteristic (ROC) curves or confusion matrices were used to compare the categorical variables (i.e., the overall diagnosis and specific classification) obtained by the expert panel and two orthopedists. The areas under the ROC curves (AUCs) of the two diagnostic methods were compared using DeLong's test (28). The Bland-Altman plots and independent t-tests were then conducted to assess the agreement of continuous variables (i.e., AI and LCEA) measured by the expert panel and two orthopedists.

The test-retest reliabilities of continuous and categorical variables were quantified by the intra-class correlation coefficient (ICC) (29) and Cohen's linear weighted kappa (30), respectively. The ICCs greater than 0.80 were considered perfect agreement (31). A kappa statistic greater than 0.80 was considered satisfactory. Statistical significance was set at *p* < 0.05. All data were statistically analyzed using SPSS 28.0 (IBM, Armonk, United States) and GraphPad Prism 9.3.1 (GraphPad, San Diego, United States).

## 3. Results

### 3.1. Diagnostic effect evaluation

**Figure 3:**
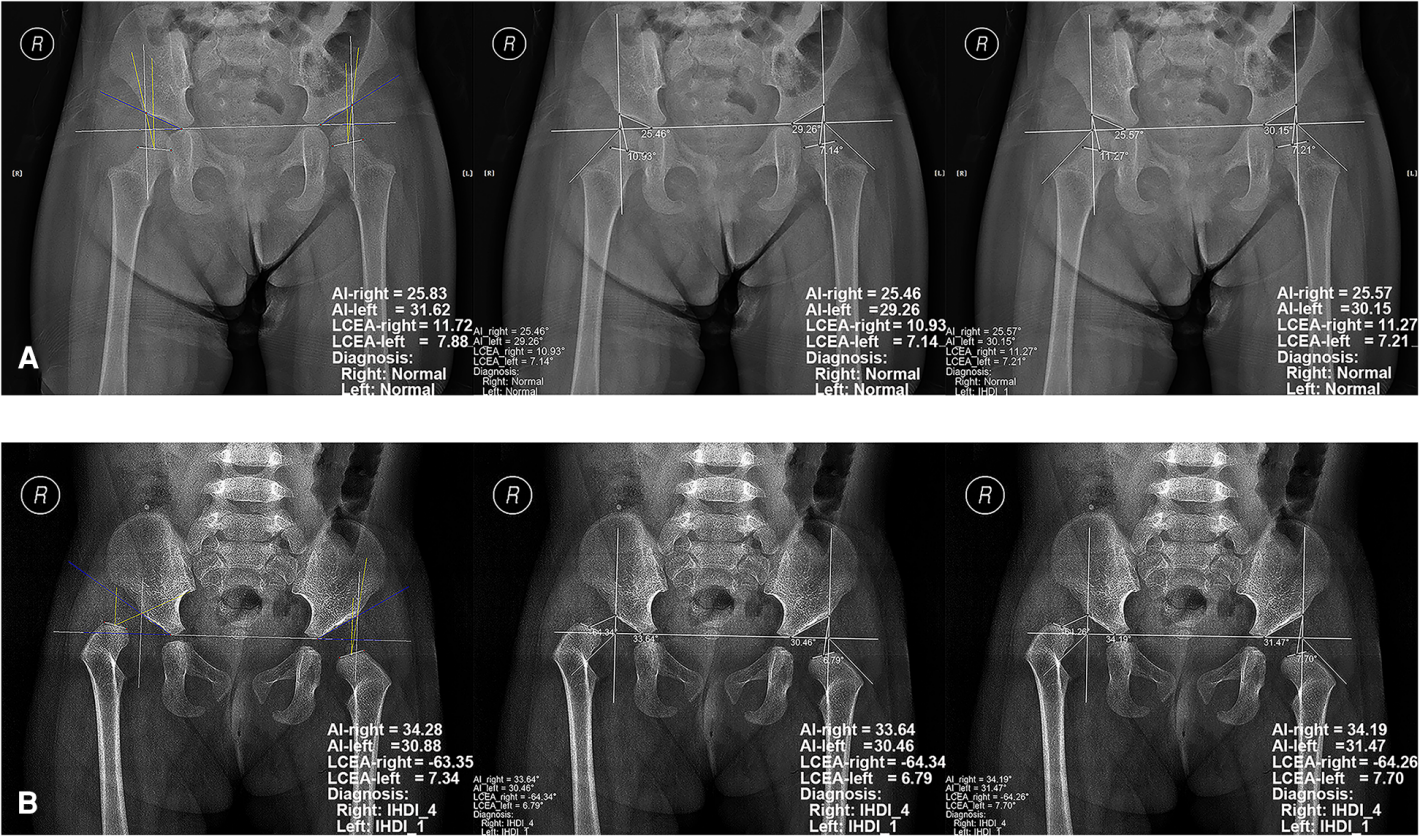
Examples of radiographic diagnosis of developmental dysplasia of the hip on pelvic radiographs by the software. The left images represent the evaluation results of the standard, while the middle and right images represent those of the software by Junior orthopedist and Intermediate orthopedist, respectively. (**A**) the pelvic radiograph of a 2.3-year-old girl; (**B**) the pelvic radiograph of a 1.4-year-old girl.

**Figure 4:**
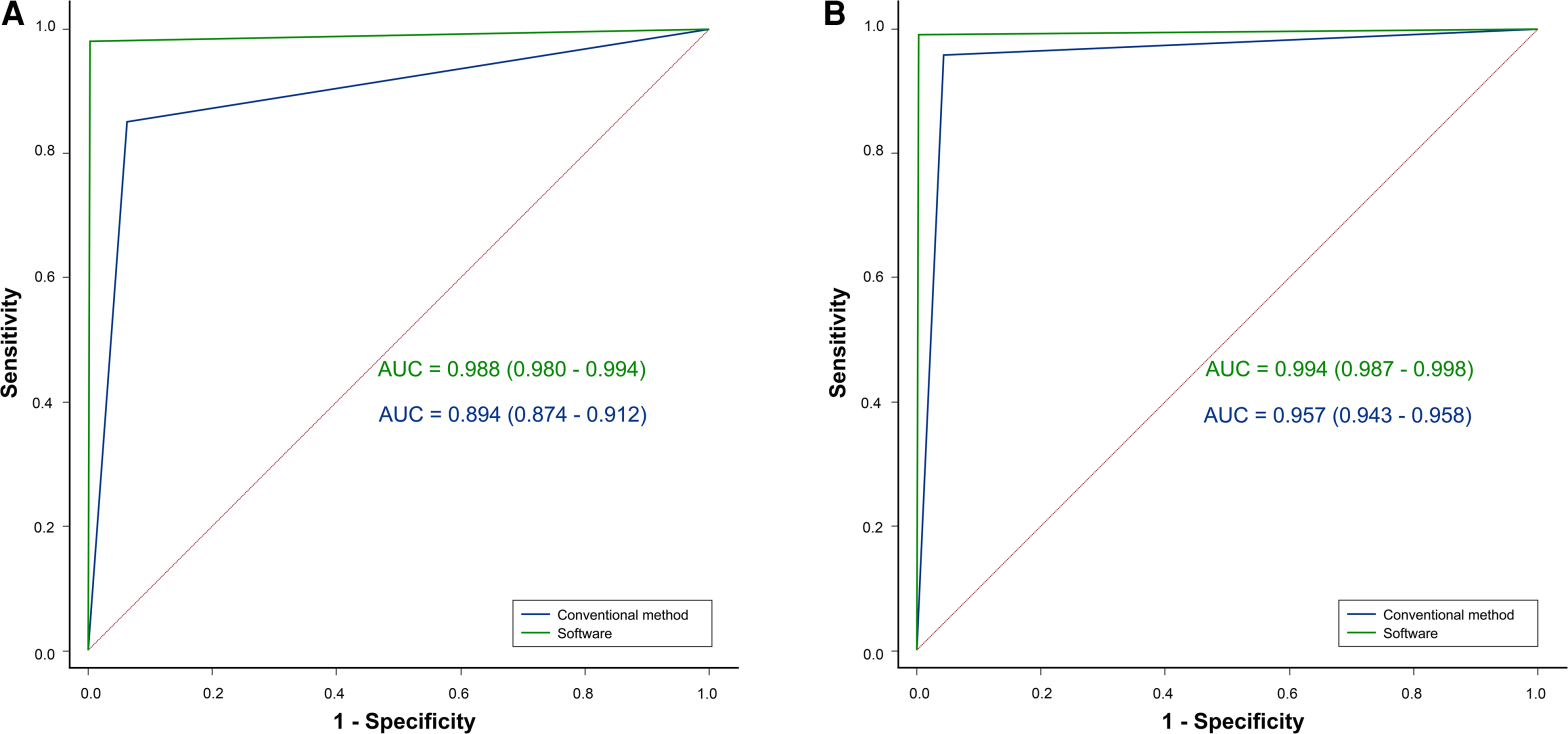
ROC analysis showing that the diagnostic performance of the software (green line) was better than that of the conventional method (blue line) in both the (**A**) junior and (**B**) intermediate orthopedists.

**Figure 5:**
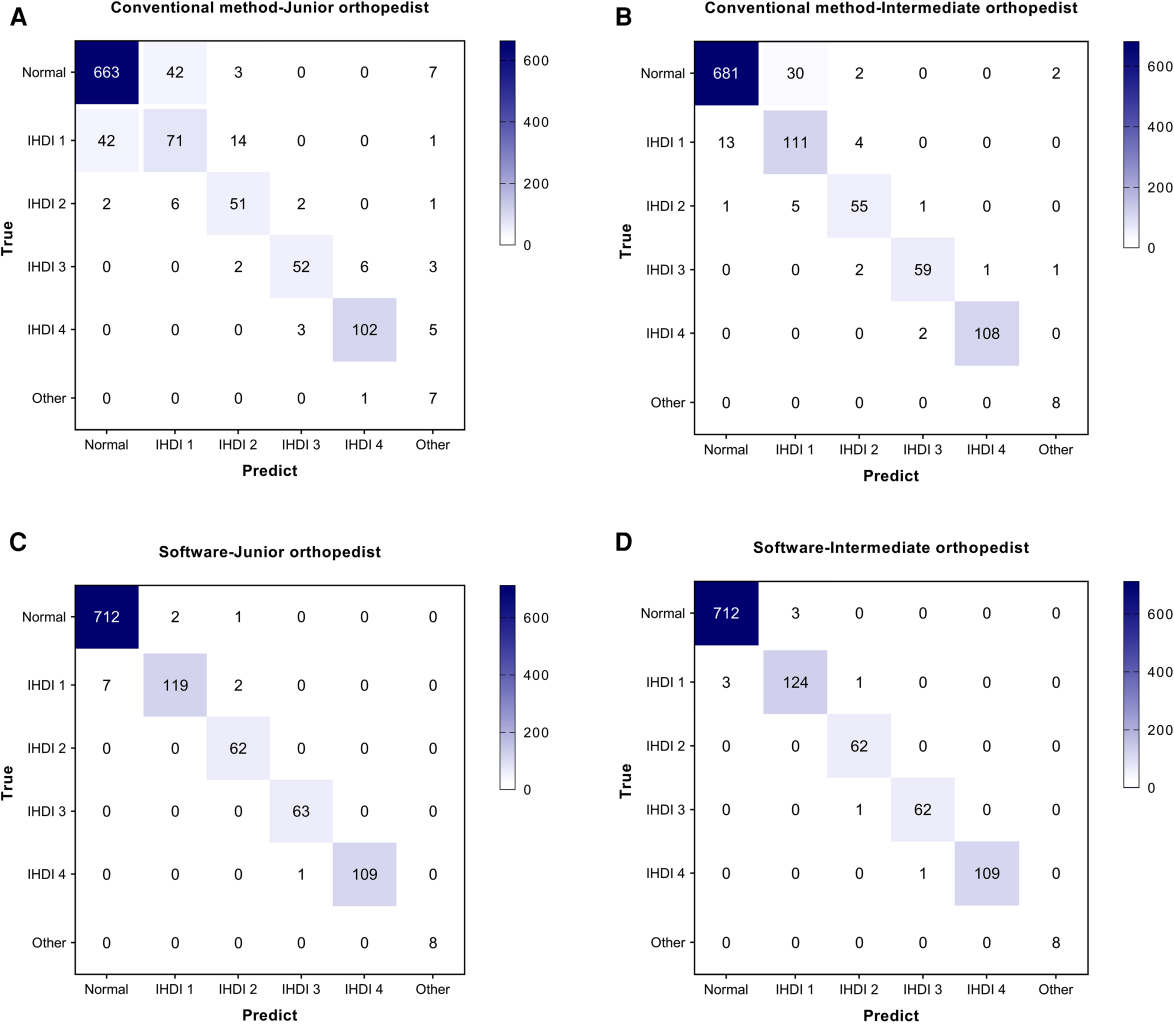
Confusion matrix of the specific classification in two orthopedists. (**A**) The confusion matrix of the conventional method by Junior orthopedist. (**B**) The confusion matrix of the conventional method by Intermediate orthopedist. (**C**) The confusion matrix of the software by Junior orthopedist. (**D**) The confusion matrix of the software by Intermediate orthopedist.

Four diagnostic indicators (AI, LCEA, overall diagnosis, and specific classification) of 543 pelvic radiographs (1,086 hips) were compared by two independent orthopedists using the software and conventional method. Selected examples of the software for diagnosing DDH are shown in Figure 3. In the conventional group, the AUC, accuracy, sensitivity, and specificity of the junior orthopedist were 0.894, 90.79% (986/1,086), 85.12% (309/363), and 93.64% (677/723), respectively. And these for the intermediate orthopedist were 0.957, 95.67% (1,039/1,086), 95.87% (348/363), and 95.57% (691/723), respectively. In the software group, the AUC was 0.988–0.994, accuracy was 99.08% (1,076/1,086)–99.72% (1,083/1,086), sensitivity was 98.07% (356/363)–100.00% (363/363), and specificity was 99.59% (720/723) (Figure 4). DeLong's test showed that the software was more effective than the conventional method in diagnosing DDH (*p* < 0.001). Moreover, the confusion matrix shows the diagnostic performance of the specific classification on the software was similar to the standard (Figure 5).

**Figure 6:**
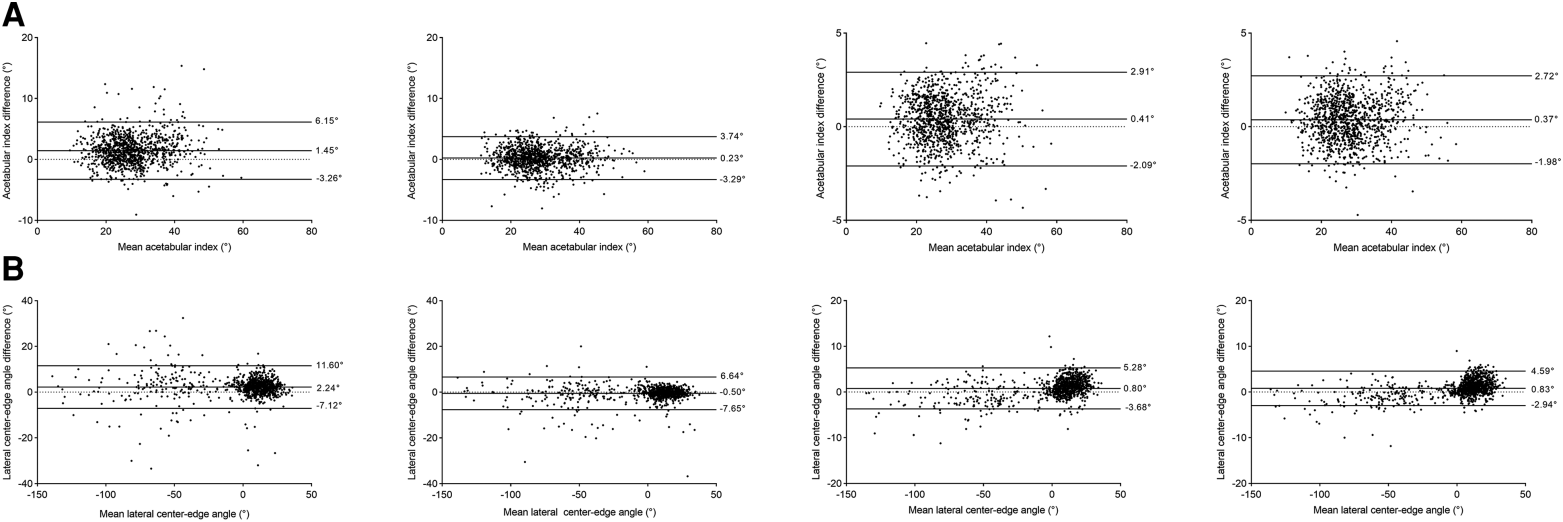
Diagnostic performance of two methods for parametric measurements using Bland-Altman plots. Two images on the left represent the diagnoses of the conventional group (for junior and intermediate orthopedists, respectively), whereas two images on the right represent the diagnoses of the software group (for junior and intermediate orthopedists, respectively). (**A**) acetabular index. (**B**) lateral center-edge angle.

The distribution of hip parameters measured by two orthopedists compared to the standard is shown in Figure 6. In the conventional group, the 95% limits of agreement (Bland-Altman analyses), for AI measurements, in the junior and intermediate orthopedists were −3.26°–6.15° (bias 1.45°, *p* = 0.522) and −3.29°–3.74° (bias 0.23°, *p* = 0.509), respectively. For LCEA measurements, the 95% limits of agreement were −7.12°–11.60° (bias 2.24°, *p* = 0.079) and −7.65°–6.64° (bias −0.50°, *p* = 0.690), separately. In the software group, as for AI measurements, the 95% limits of agreement were −2.09°–2.91° (bias 0.41°, *p *= 0.216) and −1.98°–2.72° (bias 0.37°, *p *= 0.264), respectively. With regards to LCEA measurements, the 95% limits were −3.68°–5.28° (bias −0.80°, *p* = 0.522) and −2.94°–4.59° (bias −0.83°, *p* = 0.509), separately.

### 3.2. Test-retest agreement

In the conventional group, ICC values were 0.929–0.952 (AI) and 0.986- 0.993 (LCEA), and kappa values were 0.817–0.844 (overall diagnosis) and 0.904–0.919 (specific classification), while in the software group, they were 0.936–0.953, 0.990–0.992, 0.926–0.939, and 0.956–0.965, respectively. Supplementary Figure S1 shows the perfect test-retest agreement about four diagnostic indices in the software group.

### 3.3. Diagnostic time

In this study, the time consumption of assessing the pelvic radiograph included loading the image data into the proposed software or Digimizer, plotting reference lines and points, and obtaining four diagnostic indices of pediatric DDH. For each case of 543 radiographs, the mean time consumption of the software (junior orthopedist, 10.70 ± 0.68 s; intermediate orthopedist, 10.66 ± 0.70 s) was significantly less (*p* < 0.001) than that of the conventional method (junior orthopedist, 177.77 ± 8.42 s; intermediate orthopedist, 156.62 ± 7.40 s) (Supplementary Figure S2).

## 4. Discussion

Early and accurate diagnosis of pediatric DDH is closely related to favorable treatment outcomes (32). Once diagnosed with DDH, treatment should be prompt to avoid serious consequences (33). Clinicians usually resort to medical image analysis because of the atypical symptoms of DDH in young children. Numerous auxiliary approaches have been developed recently to help clinicians diagnose DDH (9, 11, 34). These approaches focused on measuring hip parameters and determining the degree of hip dislocation. However, the clinical expert's diagnosis of pediatric DDH on pelvic radiographs requires a comprehensive analysis of whether hip parameters are abnormal by considering patients' personal information.

Compared to the experts, existing diagnostic tools lack a diagnostic strategy for DDH (9, 11, 14, 15, 25). Meanwhile, these tools may have limitations in the radiographic diagnosis of DDH. They seem to have overlooked other dislocated hips, such as hip arthochalasis and neurogenic dislocation of the hip (NDH) (35). Therefore, they were difficult to be promoted as practical diagnostic methods. In our software, the diagnostic results of pelvic radiographs were divided into normal, dysplastic, and other dislocated hips.

Previous research has revealed that the errors of inter- and intra-observer measurements in AI varied from ±3.5° to ±6.1° (36–38). These errors may be increased due to the influence of pelvic malpositioning in three-dimensional planes on the projected image (39, 40). Even artificial intelligence-assisted methods could have about ±5° errors in AI values (9, 11, 15). By contrast, the 95% limits of agreement of AI for the proposed software were −2.09°–2.91° (bias 0.409). Our results demonstrate that the software accurately diagnosed DDH in 1,076–1,083 out of 1,086 hips (99.08%–99.72%). Overall, three cases (3 hips) were diagnosed as DDH by the software but not by the expert panel. In two of these hips, detecting the lateral acetabular edge was challenging because of potential pelvic tilt. Another hip, with a high-normal AI, was misdiagnosed with DDH due to the software's measurement error. Besides, in the software group, two (3 hips) and six cases (7 hips) were misdiagnosed with normal hips by the intermediate and the junior orthopedist, respectively. Two cases (3 hips) were borderline dysplastic hips with mild abnormal AI (Supplementary Figure S3). The other four false-negative cases (4 hips) were difficult to locate the top of the triradiate cartilage due to pelvic rotation and tilt. Therefore, the hip MRI would be recommended in some borderline cases to investigate the acetabular development.

Despite numerous hip parameters, there is no universal diagnostic strategy for pediatric DDH (41). Previous studies revealed that DDH began with a shallow acetabulum and anteverted femur (8, 42). Sherman et al. found AI was an objective and preferred index for diagnosing hip dysplasia in children under eight (43). Davila-Parrilla et al. observed AI was a reliable parameter for assessing acetabular morphology (41). Novais et al. highlighted AI as a golden indicator to determine the severity and prognosis of DDH (44). However, AI is an age-dependent index as the child's pelvis gradually develops (17). Tönnis and Zhao et al. found that AI values were influenced by the patient's nationality, age, gender, and bilateral difference (17, 18). Thus, the present study used AI to assess acetabular development and considered those factors while diagnosing DDH.

Additionally, a specific diagnosis of DDH requires further evaluation of the acetabular-head relationship. The femoral ossific nucleus exhibited significant differences among children under three due to incomplete ossification (20). This phenomenon was more evident in DDH because of the delayed appearance and eccentricity of the ossific nucleus (42). In contrast to the Tönnis method, the IHDI classification relies on the midpoint of the proximal femoral metaphysis, which can be applied to children of all ages (27). Therefore, the IHDI classification was used to evaluate the severity of DDH.

### 4.1. Limitations

There are some limitations of the current study. First of all, there were a limited number of radiographs used in this study. Although only 543 radiographs were available, the proportion of cases with different diagnostic types and various age groups was reasonable. Secondly, the diagnostic strategy adopted in this study was affected by the triradiate cartilage. Therefore, the software may not be generalized to children with a history of hip surgery or premature closure of triradiate cartilage. In addition, the proposed software could not screen standard pelvic radiographs (i.e., detecting potential pelvic malpositions). In the next version, the software will be added to the quality assessment of radiographs based on Tönnis' pelvic rotation and tilt criteria (45). Lastly, the software is a semi-automatic diagnostic tool for pediatric DDH. Although this software can help clinicians significantly improve the diagnostic accuracy of DDH, it is still user-dependent. In the subsequent research, the existing deep-learning algorithms will be optimized and integrated with this tool to develop a full-intelligent comprehensive tool for diagnosing pediatric DDH.

### 4.2. Conclusion

In summary, this study suggests the software can provide expert-like analysis of pelvic radiographs and obtain the radiographic diagnosis of pediatric DDH with great consistency and efficiency. Its initial success lays the groundwork for developing a full-intelligent comprehensive diagnostic system of DDH.

## Data Availability

The original contributions presented in the study are included in the article/**Supplementary Material**, further inquiries can be directed to the corresponding author/s.
