# Supplementary material for: Clinical thought-based software for diagnosing developmental dysplasia of the hip on pediatric pelvic radiographs
Source: Front Pediatr. 2023 Mar 30;11:1080194. doi: 10.3389/fped.2023.1080194 (PMC10098126; doi:10.3389/fped.2023.1080194)
Supplement: Supplementary file 1 [file Datasheet1.pdf]

Supplementary Material

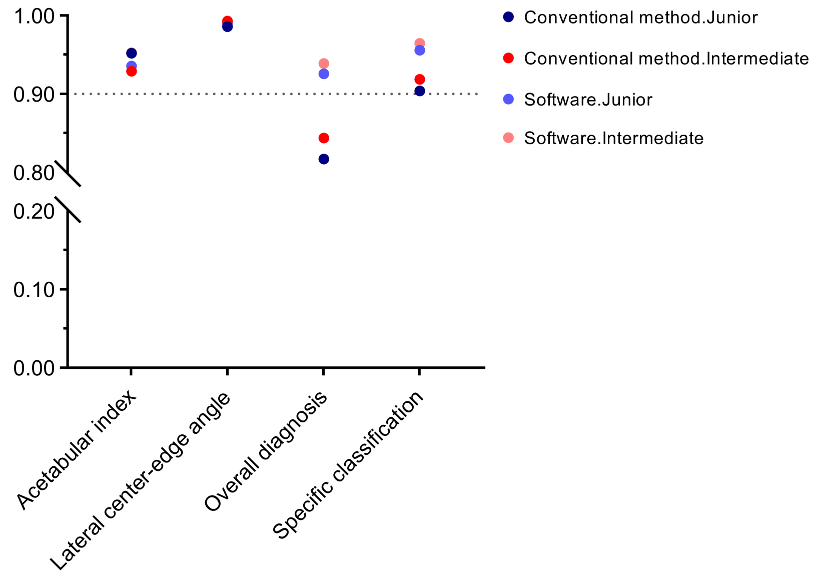

Supplementary Figure S1. The test-retest agreement of four indicators in the software and the conventional method.

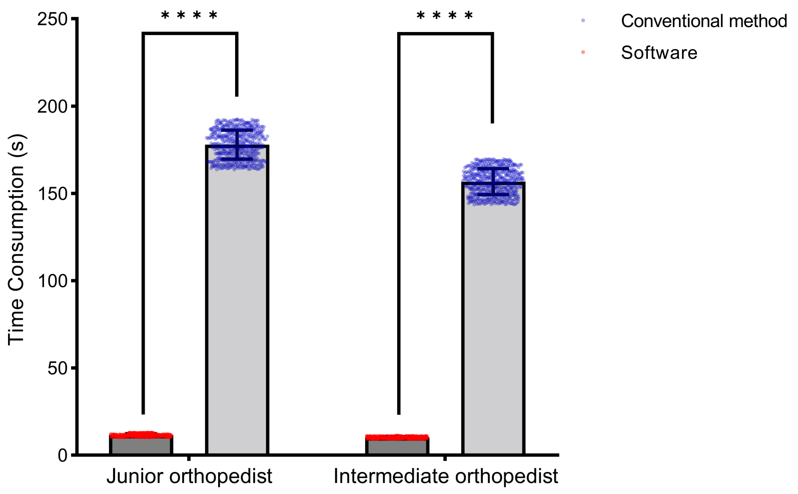

Supplementary Figure S2. Scatter graph with bars comparing diagnostic time consumed by the conventional and software group ($p < 0.0001$).

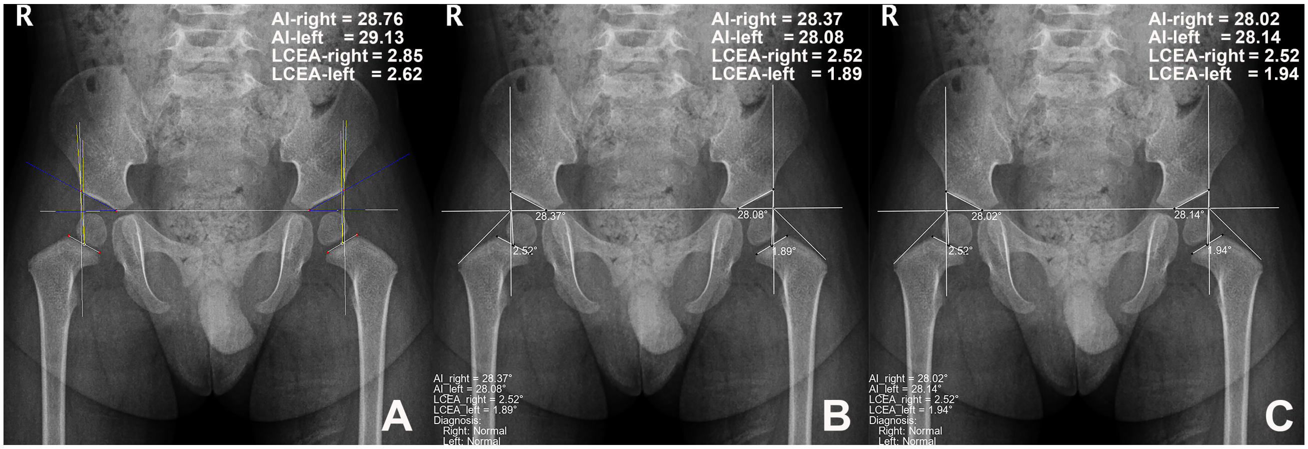

Supplementary Figure S3. A case with borderline dysplastic hips (male, 1.3 years) misdiagnosed as 'normal hips' by the software due to underestimating the acetabular index (AI). **(A)** Radiographic diagnosis of the expert panel. **(B)** Radiographic diagnosis of the software by Junior orthopedist. **(C)** Radiographic diagnosis of the software by Intermediate orthopedist.
